# Microbiome modulation in regeneration in jellyfish

**DOI:** 10.1371/journal.pone.0355863

**Published:** 2026-08-21

**Authors:** Aki Ohdera, Matthew Wang, Maille Mansbridge, Changhua Yu, Ryan Ginn, Hannah DeMerit, Isabella Guillen, Christy Lam, Andrew Hyunsoo Kim, Gianni Notaro, Jonathan Ong, David Herman, Lea Goentoro

**Affiliations:** 1 Division of Biology and Biological Engineering, California Institute of Technology, Pasadena, California, United States of America; 2 University of Pennsylvania, Philadelphia, Pennsylvania, United States of America; 3 Alverno Heights Academy, Sierra Madre, California, United States of America; 4 Flintridge Preparatory School, La Cañada Flintridge, California, United States of America; 5 Santa Ana High School, Santa Ana, California, United States of America; 6 Pasadena High School, Pasadena, California, United States of America; 7 Polytechnic School, Pasadena, California, United States of America; 8 Blair High School, Pasadena, California, United States of America; Tsinghua University, CHINA

## Abstract

The moon jelly *Aurelia coerulea* does not typically regenerate appendages. A previous study found that increasing nutrient availability promotes the activation of appendage regeneration. In this study, we found that changes in nutrient availability alter microbiome composition. Genome-scale metabolic modeling of the microbial symbionts suggests that the microbiome modulation reduces the abundance of a symbiont that competes with the host for metabolic resources. Antibiotic treatment that recapitulates the microbiome shift significantly promotes regeneration. Transcriptomic analysis of the host shows an upregulation of genes involved in the arginine biosynthetic pathway, including *argininosuccinate synthase* and *carbamoyl phosphate synthase*. Exogenous arginine supplementation promotes appendage regeneration. These findings suggest that microbiome composition, influenced by nutrient availability, contributes to the activation of host regeneration, and identify a role for arginine in promoting regeneration.

## Introduction

Recovering injured organs is energetically demanding. It is increasingly appreciated that a limiting factor in regeneration is nutrient availability. Ctenophores cease regenerative processes under nutrient-limited conditions [1]. Salamanders reared in low nutrient conditions show substantially reduced limb regeneration [2]. Nutrients are not only necessary for supporting normally occurring regeneration; increasing nutrient availability can promote activation of regeneration in poorly regenerating systems. In jellyfish, increasing nutrient availability promotes activation of appendage regeneration [3]. In tadpoles, which stop regenerating their tails during the refractory period, increased feeding restores tail regeneration [4]. In adult mice, which do not normally regrow digits from proximal amputations, increasing nutrients promotes activation of digit regrowth [3]. Given the role of nutrients in regeneration, we investigated whether the microbiome plays a role in mediating this relationship.

The microbiome can profoundly influence the host nutrient metabolism [5]. The microbiome can aid the breakdown of nutrients, produce molecules otherwise not available to the host, and increase amino acid availability [e.g., 6–10]. From flies to mice, the microbiome can compensate for undernutrition and rescue growth [11,12]. In parallel, the microbiome has also been linked to regenerative processes [13]. In planarians, a pathogenic shift in the microbiome inhibits regeneration [14]. In mice, taxa-specific signals from the gut influence regeneration processes in remote muscle and liver [15]. In frog tadpoles, antibiotic treatment inhibits tail regeneration, which can be rescued by supplying lipopolysaccharides [16]. While these studies connected microbiome and regeneration, few have looked into this connection in the context of varying nutrients. In this study, we began exploring the connections between nutrients, microbiome, and regeneration.

**Fig 1:**
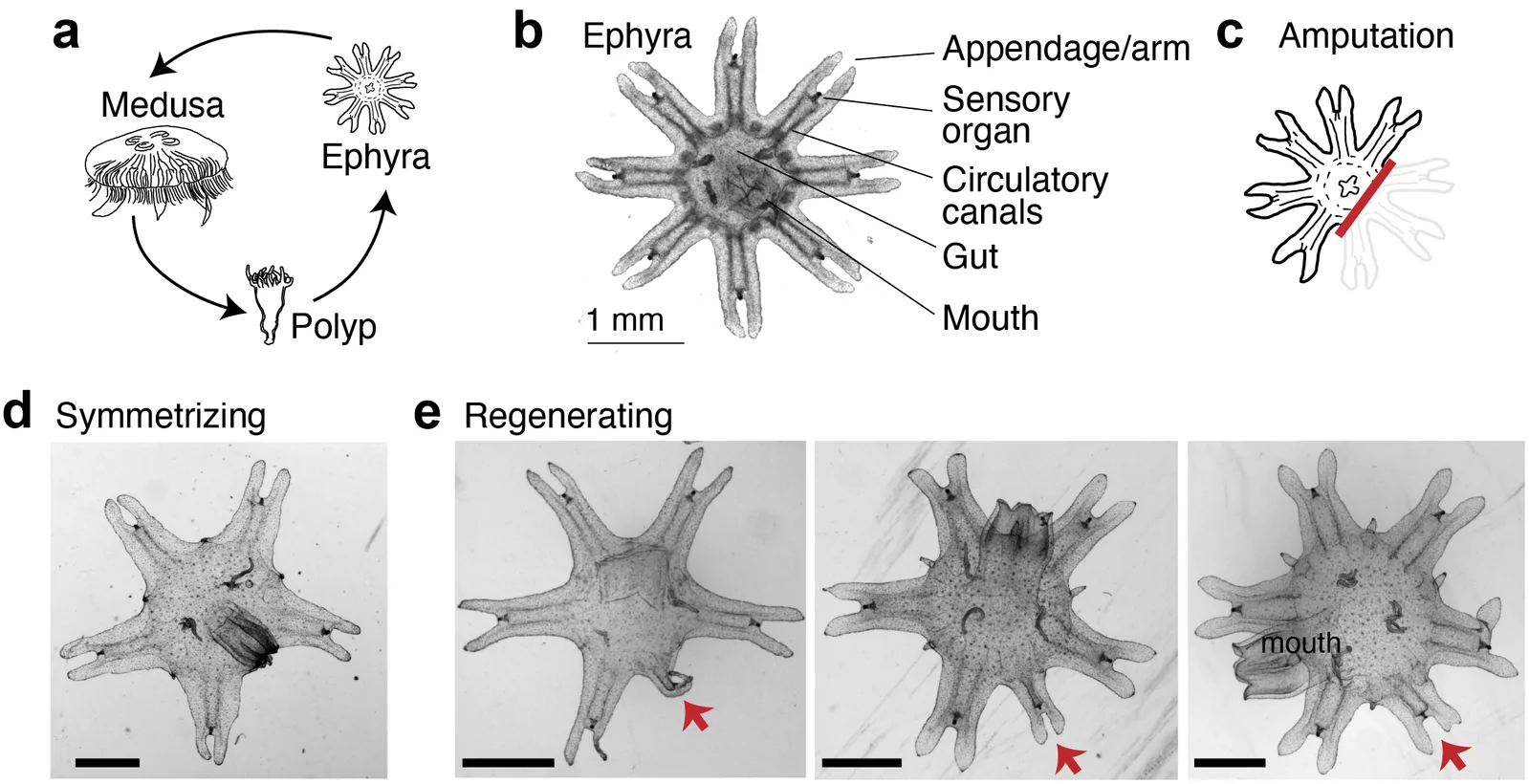
Increasing nutrient availability promotes activation of regeneration in moon jelly. **(a)** The moon jelly has a dimorphic life cycle. Sessile polyps reproduce asexually or, with favorable environmental cues, metamorphose into free-swimming juveniles, called ephyrae. Ephyrae grow into sexually reproductive medusae in typically 1–3 months. **(b)** The experiments in this study were performed on ephyrae, whose eight discrete swimming arms facilitate assessment of regeneration. **(c-e)** Ephyrae were amputated across the body (red line), removing three arms **(c)**. Injured ephyrae typically reorganize existing arms and regain symmetry within 1–3 days **(d)**. With increasing nutrient availability, as will be reproduced later, some ephyrae begin to regrow partial arms **(e)**. Red arrows denote the arm regenerates. Regeneration remains partial even at 2–3 weeks after amputation, around which time, as part of the transition to medusa stage, bell tissues begin to develop between the arms, which complicates assessing further arm regrowth. Scale bar: 1 mm.

To investigate this, we used the moon jellyfish *Aurelia coerulea* as a model system. *Aurelia* is well-suited for this study because its regeneration responses vary across its life cycle (Fig 1a), allowing us to assay regeneration induction. In the sessile stage, similar to other cnidarian polyps such as hydras and sea anemones, *Aurelia* polyps readily regenerate body parts [17,18]. However, in the free-swimming stages, *Aurelia* ephyrae and medusae show limited regeneration [19,20]. This is clearly demonstrated in the ephyra (Fig 1b), whose eight discrete appendages, called arms, facilitate tracking of regeneration, or the lack of it. These arms function in swimming and feeding; they contract synchronously to produce fluid flow that propels the animal and captures prey [21]. In response to amputation (Fig 1c), *Aurelia* ephyrae rapidly reorganize the remaining body parts and arms, and regain radial symmetry (Fig 1d). The recovery of radial symmetry facilitates propulsion and feeding, and ultimately, continued survival [19]. While symmetrization is the primary response to injury, as noted above, it was found that increasing nutrient availability promotes ~40% of animals to begin regenerating partial appendages (ref. 3; reproduced here in Fig 1e and again later in Fig 4b). In addition to the established connection between nutrients and regeneration, the microbiome of *Aurelia* has been characterized. A recent study found that the *Aurelia* microbiome is dominated by two bacterial taxa [22]. Therefore, *Aurelia* offers a low complexity microbiome system to interrogate how the microbiome and nutrients interact to affect regeneration in a poorly regenerating animal.

## Materials and methods

**Polyp culture** of *Aurelia coerulea* (formerly *Aurelia aurita* sp. 1) used in this study originate from specimens collected by the Cabrillo Marine Aquarium off the coast of Long Beach, CA, USA (33^o^46’.2”N 118^o^0744.2W). Polyps were maintained in 32–35 ppt artificial seawater (ASW), at 20–22^o^C, with 12:12h light cycle, and fed every other day with 48-hr old *Artemia* (Brine Shrimp Direct, BSEP1.75Z), enriched with RGComplete (Reed Mariculture). To induce metamorphosis, polyps were treated overnight with 25 μM 5-methoxy-2-methyl-indole (Acros Organics, 10434575). Treated polyps were rinsed three times with ASW and fed every other day until metamorphosis began. Metamorphosis typically occurred ~10 days after induction. Newly strobilated ephyrae were fed L-size rotifers (Reed Mariculture) for two days prior to experiments.

**Amputation experiments and treatment** were performed using an X-acto knife fitted with a #17 blade following methods described previously [3]. Under a dissecting microscope, ephyrae held in a petri dish were amputated by cutting across the body to remove three arms (Fig 1c). Two-day old ephyrae were used for the amputation experiments. Amputated ephyrae were placed in 1 liter settling cones (Nalgene Imhoff, 1000−0010), with gentle aeration, at 25–40 ephyrae/cone and 15 mL of artificial seawater/ephyra. For control, low-food condition, ephyrae were fed ~ 5 rotifers per animal every other day. For the increased food condition, ephyrae were fed ~ 40 rotifers per animal. Rotifer density was determined by calculating the average number of rotifers in five replicates of 50 ul aliquots of well mixed culture to calculate the per milliliter density. The feeding level provided here should only be used as a starting estimate, because in our experience, the response to feeding can sensitively depend on polyp batches, as reflected in the variation across experiments from independent strobilation. We verified in a previous study that this is not due to genetic variations in the population, as the same variation occurs in clonal populations [3]. Accordingly, to establish the experiments, one would need to first calibrate what is low vs high feeding level in their own lab. For penicillin treatment, penicillin was added to the water to give a final concentration of 100 U/mL (G-Biosciences, RC-741). For arginine experiments, L-arginine methyl ester dihydrochloride was added to the water to give a final concentration of 200 μM (Chem-IMPEX International Inc., 03022). Penicillin and arginine treatments were performed with control or increased food condition, as specified in the main text. Water (along with everything in it, food, penicillin, or arginine) was refreshed every other day. Regeneration was assessed at 14 days post-amputation: individual ephyra were imaged, assessed for presence of a newly regenerated arm, and length of regenerated arm quantified using Fiji.

### Statistical analysis

To assess the statistical significance in the regeneration experiments, we performed meta-analysis [23]. Meta-analysis was utilized because independent cohorts of animals (from different rounds of strobilation/hatching) can show different baseline rates of regeneration. Consequently, to accurately assess treatment effects, control and treatment groups needed to be set side-by-side using the same cohort of animals, and the treatment effect evaluated within each experiment. Meta-analysis was then performed to assess the reproducibility of the treatment effect across experiments.

Within each experiment, the treatment effect was quantified using standard metric. For the regeneration frequency data, which are in the form of 2 x 2 table, we used the Risk Ratio metric:


$$\begin{array}{c} Treatment\;effect\;size=\frac{\%\;of\;regenerating\;animals\;in\;treated\;group}{\%\;of\;regenerating\;animals\;in\;control\;group} \end{array}$$

When regeneration frequency is zero in one of the datasets being compared, following the standard procedure, the constant 1 was used to compute the effect size. For the regenerate arm length data, which are quantitative, we used the Response Ratio metric:


$$\begin{array}{c} Treatment\;effect\;size=\frac{average\;length\;of\;arm\;regenerate\;in\;treated\;group}{average\;length\;of\;arm\;regenerate\;in\;control\;group} \end{array}$$

The length is computed as percent of intact arm length within the same individuals, to account for variations of arm length across individuals. Datasets with zero regenerates in control were not included in the calculations. Having calculated the treatment effect sizes from multiple independent experiments, we used the restricted maximum likelihood model (REML), which enables taking into account heterogeneity across experiments, to assess the reproducibility of the treatment effects across experiments. The analysis was implemented using the metafor package in R ([24]; Response Ratio is called Ratio of Means in metafor).

### Quantitative PCR

Control and treated unamputated ephyrae grown as described above were collected after 7 days and flash frozen. DNA extraction was performed using the DNeasy Blood and Tissue Kit (Qiagen, 69504), with a 3 hour proteinase K treatment. qPCR was performed with the QuantiTect SYBR Green PCR Kit (Qiagen, 204143) and StepOne Real-Time PCR system (Applied Biosystems, 4376357). Species-specific primers were used to target the bacterial 16S rDNA genes:

*Mariplasma* forward: TGCGTAGATGGTGAAATTAGTC
*Mariplasma* reverse: CGCTATTGGTGTTCCTTCATA
*Marinirickettsia* forward: GTTATTTGTGAAAGCCCTAAGC
*Marinirickettsia* reverse: GCCTTCGCTGTTGGTATT

To normalize the sample load across wells, the host gene *Sox1* was used:

*Sox1* forward: CGCCTTGAAAGATAC CCTAAA
*Sox1* reverse: AGCACACCATTCTCCATTATA

Primer specificity was confirmed by gel analysis and Sanger sequencing the amplicon. The qPCR reactions were run with the following condition: an initial denaturation step at 95°C for 10 minutes, followed by 40 cycles of 95°C for 15 seconds and 60°C for 1 minute. A melt curve analysis was performed immediately after amplification to confirm product specificity.

To calculate the relative abundance of the bacteria, *Ct* values were first converted to raw DNA quantities and corrected to account for primer efficiency using the formula:


$$\begin{array}{c} DNA\;quantity={\text{1}\text{0}}^{\left((\text{Ct value}-intercept)/-slope\right)} \end{array}$$

The DNA quantity was then normalized by the host *Sox1* gene quantity, to account for loading variability. Subsequently, the *Sox1*-normalized DNA quantity was normalized by the mean of the control data. These are the relative bacterial abundances plotted in the main figures. *Mariplasma*-to-*Marinirickettsia* ratios were calculated by taking the ratio of *Mariplasma* and *Marinirickettsia* relative abundance within each biological replicate. To compute statistical significance, the non-parametric Kruskal-Wallis test was used.

**Reconstruction of the genome-scale metabolic models (GEMs)** was performed following the established workflow. First, an automated model reconstruction of the *Mariplasma* and *Marinirickettsia* genomes (NCBI Accession PRJNA975886) was performed with CarveMe (version 1.3.0) [25]. Next, the resulting model was further manually curated. Each reaction in the model was cross-referenced to the gene annotation and function. Gene functions were determined by BLASTp against the NCBI nr database. Based on the BLASTp results, reactions that fell into the following criteria were removed: (1) The reaction does not have functioning enzymatic annotation; (2) The reaction does not have a flux under optimum growth conditions (as determined with *cobra_model.optimize()*, where *cobra_model* is the name of the model input) and does not affect the feasibility of the model under a knock out simulation (using *cobra_model.reactions.reactionID.knock_out(), where cobra_model* is the name of the model input and *reactionID* is the name of the reaction); and (3) The reaction is not likely to be present for the species, as assessed from available experimental literature. The resulting, curated model was solved using Flux Balance Analysis (FBA) and Flux Variability Analysis (FVA) in COBRApy ver. 0.15.4 [26]. The models were solved to optimize the growth rate, using the default objective function. To achieve growth rates comparable to those experimentally observed for Mollicutes and Rickettsiales species (S1 Table), we varied the upper and lower bounds for metabolite uptake and secretion (for both models, the final values were, *exchange_default_ub* = 1000, *exchange_default_lb* = −10).

### RNA sequencing and analysis

Animals were amputated as described above, and then reared in three conditions: control, increased food at 3–4 times the amount of control, and control food supplemented with penicillin. At five days after amputation, 5–7 animals were flash frozen in liquid nitrogen. RNA extraction was performed using the RNeasy Plus Mini kit (Qiagen, 74104), with the following modifications. RLT buffer containing β-mercaptoethanol was added to the frozen samples and the samples were homogenized on ice. Homogenized samples were centrifuged with the QIAshredder at 12,000 g for 2 minutes, and RNA was then purified with the RNeasy kit following the manufacturer’s protocol. DNA was removed from the samples using the DNA-free DNA Removal kit (ThermoFisher Scientific, AM1906), followed by poly-A selection using NEBNext Poly(A) mRNA Magnetic Isolation Module (NEB, E7490S). Samples were submitted to the Caltech Millard and Muriel Jacobs Genetics and Genomics Core facility for library prep and 50 bp paired-end sequencing using NextSeq 2000 sequencer. Reads were trimmed and quality filtered using Trimmomatic (version 0.39; [27]). Trimmed reads were mapped to the *Aurelia coerulea* genome [28] using STAR (version 2.5.3a; [29]) to generate the counts table. Differential gene expression analysis was conducted using DESeq2 (version 1.34.0; [30]). The Wald test was used for hypothesis testing. Log2 fold change shrinkage was applied for pairwise contrasts with a false discovery rate cutoff of 0.05. Gene Ontology analysis was performed using the R package clusterProfiler (version 4.2.2; [31]), using Biological Process aspect and a q-value threshold of 0.05, and plotted using REVIGO [32].

**Presence or absence of *arginase*** across cnidarian groups was analyzed using available genomes [33–40], considering them as representative among currently annotated genomes. The cnidarian genomes were retrieved from the repositories specified in the source papers. For *Hydra*, protein models from version 2.0 were retrieved from the NHGRI repository (https://research.nhgri.nih.gov/hydra/). Gene models were queried with a representative set of arginine cycle genes from *Nematostella* (XP_032228695.1, XP_001635121.1, XP_048589032.1, XP_020896709.1, XP_001640743.2) using protein BLAST [41].

## Results

### Increasing nutrient abundance alters the microbiome composition

As characterized in previous work [22], the microbiome of *Aurelia* is dominated by two bacterial taxa, a Mollicutes (*Candidatus* Mariplasma lunae) and a Rickettsiales (*Candidatus* Marinirickettsia aquamalans). In the ephyra stage, the two bacteria encompass ~97% of the total bacterial abundance, at ~1:1 ratio. Although minor taxa may also contribute to host physiology, our analysis focused on the two dominant taxa.

**Fig 2:**
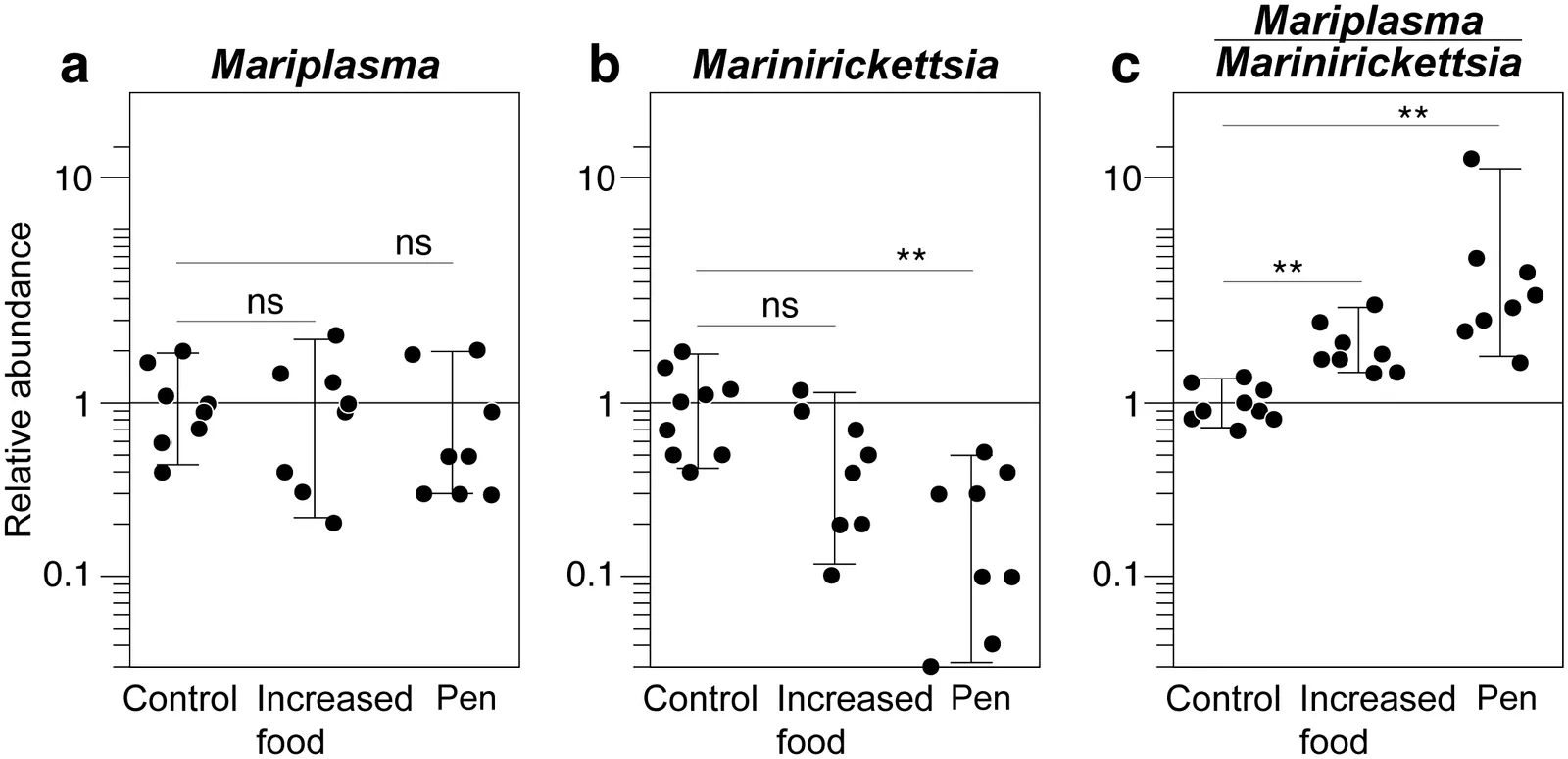
Increasing nutrient availability or treatment with penicillin shifts microbiome composition, toward reducing the relative abundance of *Marinirickettsia.* Three conditions were assessed: control level of feeding (see Methods for amount and frequency), increased level of food (~eight times higher than control food), and control food supplemented with 100 U/mL penicillin (Pen). Ephyrae were collected seven days after the start of the treatment. Quantitative PCR was performed using species-specific primers against the *16S rRNA* genes. Each bacterium only has a single copy of a 16S *rRNA* gene. Each data point (black circle) is a measurement from a biological replicate (25 animals). From each biological replicate, we measured the relative abundance of *Mariplasma* **(a)**, *Marinirickettsia* **(b)**, and the ratio between two **(c)**. Within each plot, the data are normalized to the average of the controls. Statistical analysis: Kruskal-Wallis test. ns: not significant. ** p < 0.001.

To quantify these taxa, we utilized the metagenome-assembled genomes from our previous study [22] to design species-specific primers for quantitative PCR analysis (Fig 2). Measurements of the relative abundance of *Mariplasma* and *Marinirickettsia* in the control condition are shown in Fig 2a and 2b, respectively. Although the abundance of *Mariplasma* and *Marinirickettsia* vary across biological replicates, their relative composition within each biological replicate is more precise, as seen in the narrower spread of the *Mariplasma*-to-*Marinirickettsia* ratio (Fig 2c). Note that the designation of “control” feeding level is for simplicity, and does not presume the nutrient level in the wild. Nutrient level is likely to vary in the wild; we chose as the control a feeding level in the lab that best recapitulates a typical growth rate in wild populations (ephyrae mature to medusae over 1–3 months; [42]).

Having established the assay, we examined the effect of increasing nutrient availability. Increasing food does not affect *Mariplasma* abundance (Fig 2a). Increasing food also does not affect *Marinirickettsia* abundance with statistical significance, although there is a noticeable downward shift in the spread (Fig 2b). This downward shift in *Marinirickettsia* abundance leads to a statistically significant increase in the *Mariplasma*-to-*Marinirickettsia* ratio (Fig 2c, by a mean of two-fold; p < 0.001; Kruskal-Wallis test, Pen vs Control). These measurements show that the nutrient environment modulates microbiome composition.

### Genome-scale metabolic reconstruction of *Marinirickettsia* and *Mariplasma*

**Fig 3:**
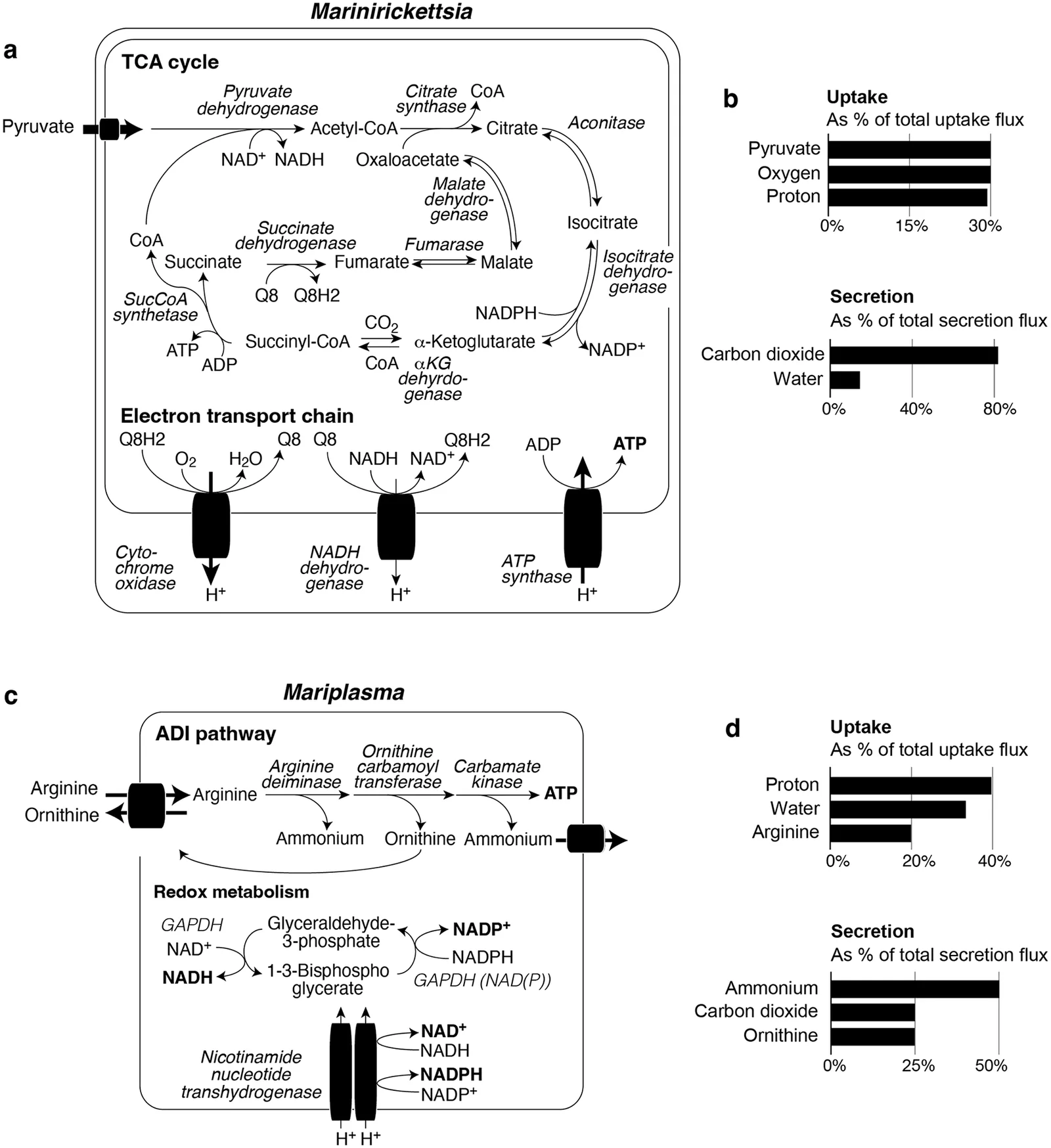
Genome-scale reconstruction of the bacterial metabolic networks. **(a, c)** Each GEM diagram shows the reactions that form the top 90% of the total fluxes in the system. See S1 Table for a complete list of the GEM reactions and fluxes. In the *Marinirickettsia* GEM, Q8: Ubiquinone-8, Q8H2: Ubiquinol-8. **(b, d)** Shown are metabolites whose transport fluxes form the top 90% of the uptake and secretion. For instance, in the *Marinirickettsia* GEM, uptake of pyruvate constitutes 30% of all uptake fluxes **(b)**; while in the *Mariplasma* GEM, ornithine secretion forms 25% of all secretion fluxes from the bacterium **(d)**. The full GEM solutions can be found in S1 Table.

The impacts of nutrient availability on the microbiome composition motivated us to build genome-scale metabolic models (GEMs) of the two dominant bacterial taxa (Fig 3). A GEM translates the genomic information into a network of metabolic reactions, which have proven to be a useful approach to assess biological capabilities of novel bacteria [43]. We built GEMs using the metagenome-assembled genomes of the bacteria [22]. Facilitating the GEM analysis is the small genomes of the bacteria (0.4 Mb for *Mariplasma*, 1 Mb for *Marinirickettsia*). The small genomes and consequently reduced numbers of genes mean that there are few alternative metabolic networks that the genome enables. We verified that the GEM solutions are robust to variation in parameters (S1 Fig).

The *Marinirickettsia* GEM (Fig 3a) is dominated by fluxes of the tricarboxylic acid cycle (TCA), which accounts for 33% of the total fluxes, and electron transport chains, which account for an additional 39%. *Marinirickettsia* GEM does not possess a functioning glycolysis pathway. Consequently, to fuel its TCA cycle, *Marinirickettsia* is predicted to uptake pyruvate (Fig 3b). In addition to pyruvate as the top uptake, *Marinirickettsia* is predicted to sequester from the host a multitude of other metabolites, including the majority of amino acids and cofactors (S1 Table). In contrast to *Marinirickettsia* GEM, the *Mariplasma* GEM, while also predicted to uptake some metabolites from the host, also secrete nitrogenous metabolites (Fig 3c and 3d). Growth in the *Mariplasma* GEM is fueled by the arginine deiminase (ADI) pathway, whose fluxes account for 71% of total flux in the model (Fig 3c). The ADI pathway is conserved across multiple bacterial taxa [44]. It metabolizes arginine to produce ATP, and generates as by-products, ammonium and the amino acid ornithine. Since the *Mariplasma* genome encodes no genes for arginine biosynthesis, the GEM predicts that the bacterium uptakes arginine from the host (Fig 3d). Almost the entire arginine taken up (98%) is used to fuel the ADI pathway, with the remaining 2% assimilated for growth, consequently predicting secretion of ornithine and ammonium (Fig 3d).

Thus, the GEM analysis shows that the bacterium that is depleted in the regenerating condition, *Marinirickettsia*, is predicted to sequester high-energy metabolites from the host. On the other hand, *Mariplasma*, which remains unaffected in the regeneration condition, is predicted to sequester fewer metabolites from the host.

### Reducing *Marinirickettsia* with an antibiotic promotes host regeneration

Based on the qPCR results and the GEM predictions, we tested whether reducing *Marinirickettsia* impacts host regeneration. *Mariplasma* belongs to the class Mollicutes, which is characterized by the lack of a cell wall [45]. To selectively target *Marinirickettsia*, we therefore tested penicillin that disrupts cell wall synthesis.

**Fig 4:**
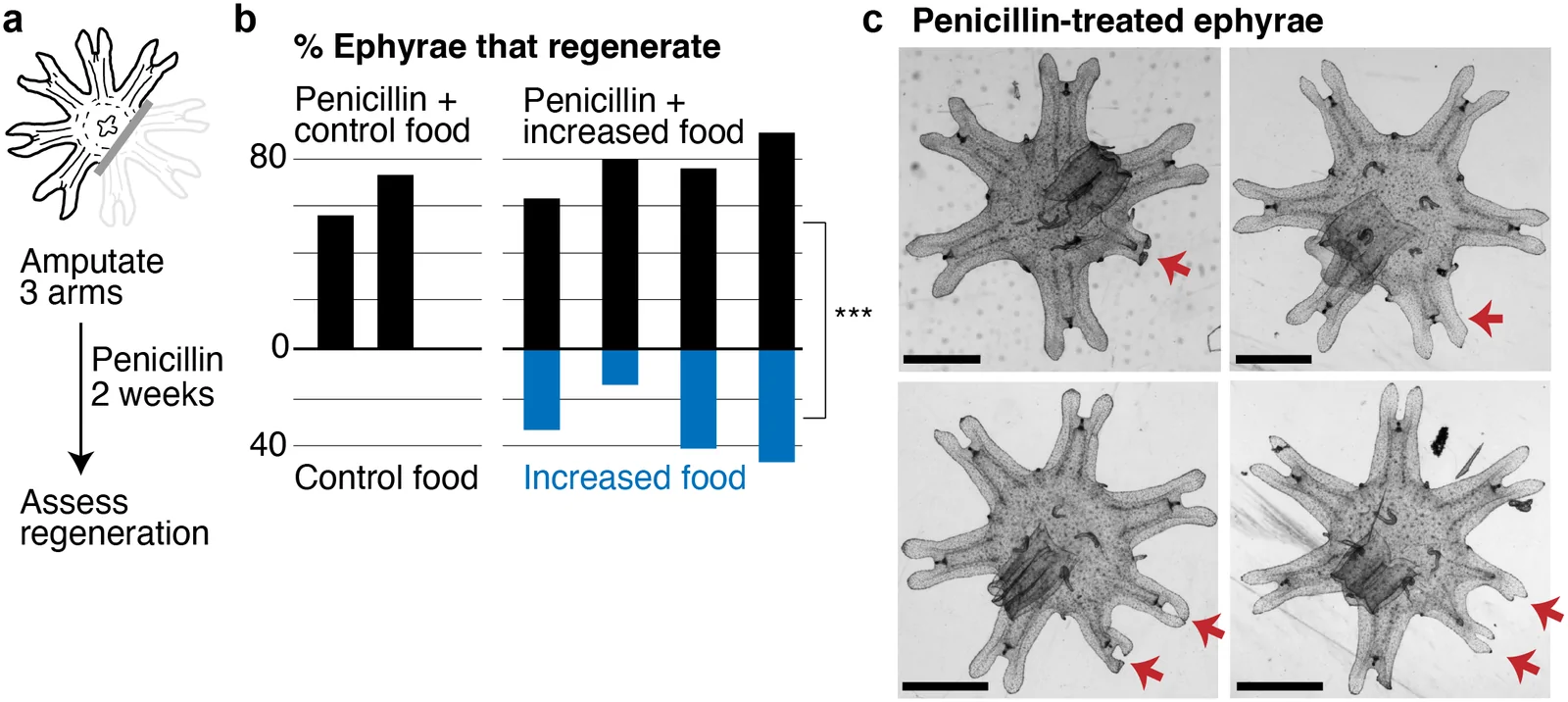
Penicillin treatment promotes regeneration. **(a-b)** Ephyrae were amputated across the body (grey line) to remove three arms, and placed in regular seawater or seawater mixed with penicillin at 100 U/mL. Feeding was performed at control level or increased level, at 3–4 times higher than control. Regeneration was assessed two weeks after amputation. Each top and bottom pair of bars is an independent experiment, in which the two groups of animals were set up side by side (20–115 animals pooled in each group). Statistical significance: meta-analysis. ***p < 0.0001. The data used for the plot can be found in S2 Table. **(c)** Representative ephyrae in penicillin treatment. Red arrows: regenerating arms. Scale bar: 1 mm.

Penicillin treatment, as expected, does not affect *Mariplasma* abundance (see ‘Pen’ in Fig 2a). By contrast, penicillin treatment produces a marked decrease in *Marinirickettsia* (by 5-fold, Fig 2b), which correspondingly shifts the *Mariplasma*-to-*Marinirickettsia* ratio (Fig 2c). Next, to assess the effect of reducing *Marinirickettsia* on host regeneration, we performed amputation experiments and treated the amputated animals with penicillin (Fig 4a). Penicillin treatment strongly promotes regeneration (Fig 4b and 4c, and see more replications in S2 Table; p < 0.0001; meta-analysis, Pen vs Control): 65–75% of penicillin-treated animals showed regeneration activation even when the control group showed none (left panel in Fig 4b). Moreover, penicillin treatment shows an additive effect with increasing nutrients (right panel in Fig 4b). Although regeneration frequency varies from clutch to clutch, as also previously observed [3], penicillin treatment consistently increases the frequency of regeneration activation. Finally, penicillin treatment not only increases the frequency of regeneration, penicillin-treated animals can regenerate longer arms (morphometric analysis in S2 Fig).

### Regenerating animals upregulate genes related to arginine metabolism

**Fig 5:**
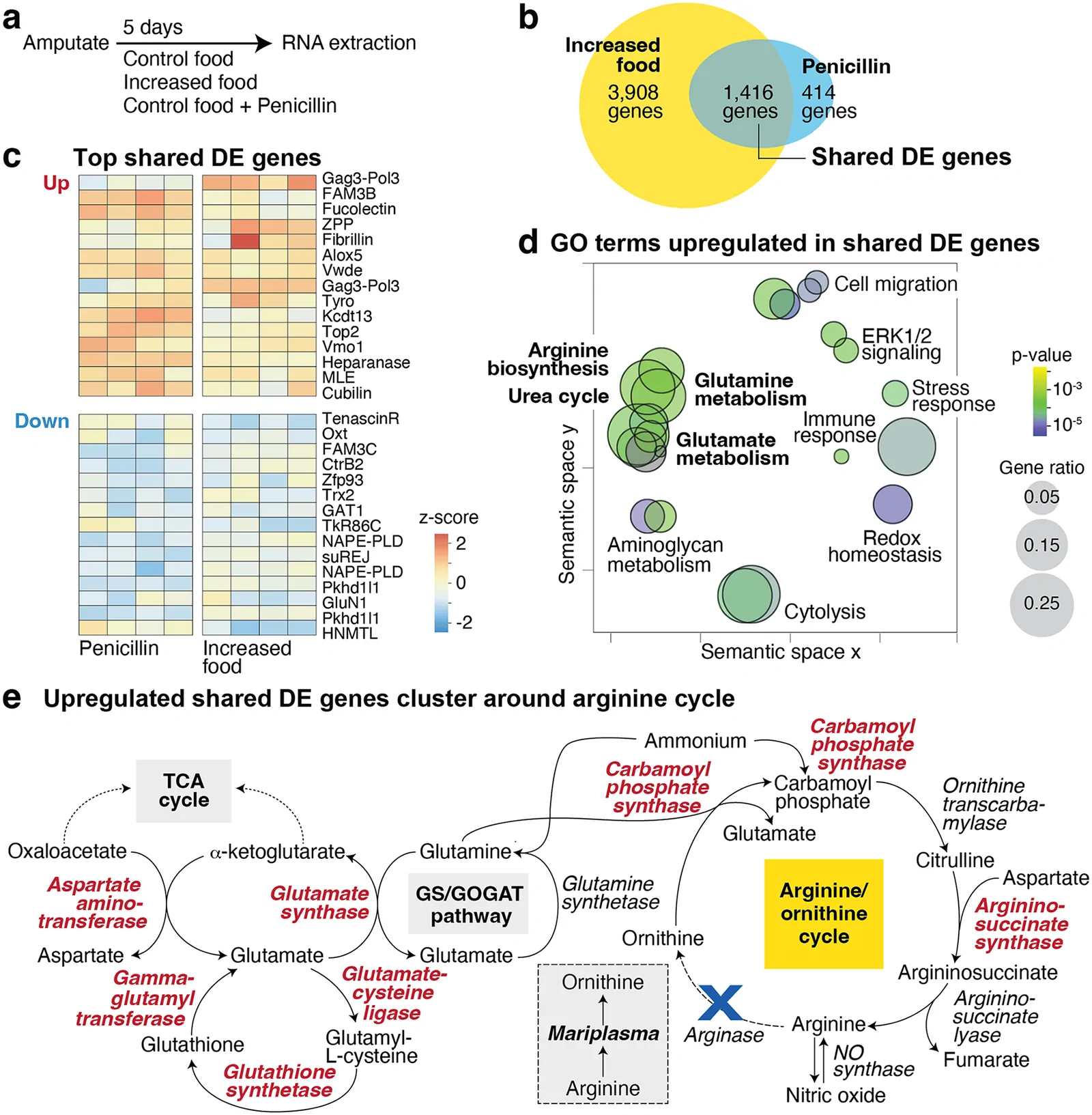
Arginine metabolism is upregulated in the regenerating hosts. **(a)** Three conditions were tested: control food, increased food at eight times higher the control amount, and control food supplemented with 100 U/mL Penicillin. For each condition, four biological replicates were collected (6–15 animals in each). **(b-c)** Differentially expressed (DE) genes were determined using DEseq analysis. Statistically significant DE genes are those with p-value <0.05 in both pairwise comparisons (Increased food vs Control and Pen vs Control). **(c)** Color indicates z-score, the regularized transcript counts for each gene in log2 scale. Only annotated genes are shown in the heat map; see S3 Table for the full list of DE genes. **(d)** GO terms enriched in upregulated shared DE genes, plotted using Revigo. A q-value cutoff of 0.05 was used for the GO enrichment analysis. Circle size: GeneRatio, i.e., ratio of number of DE genes to total number of genes in the GO term. Circle color: statistical significance of the enrichment. **(e)** This diagram is based on the KEGG arginine biosynthesis pathway, curated for those genes that we have verified as present in the *Aurelia* genome. Arrow indicates an enzymatic reaction; enzyme catalyzing the reaction is shown in italics. Enzymes shown in ***red italics*** are upregulated. Blue cross: *arginase* is missing from the *Aurelia* genome (S3 Fig), but intriguingly, as suggested in the dashed box, *Mariplasma* can in theory complement this missing biochemical link; more on this in Discussion.

Having analyzed the microbial side, we next investigated changes in the host transcriptional activities under regeneration-promoting conditions. To do this, we performed RNA sequencing (RNAseq). We compared three conditions: the low-regeneration condition (control food) and the two high-regeneration conditions (increased food and penicillin; Fig 5a). We analyzed two regeneration-promoting conditions because we reasoned that while each treatment may have non-specific effects, the overlap between the two would give a better assessment of regeneration-specific processes. Animals were collected five days after amputation for RNA extraction, just prior to onset of morphogenesis, to focus the analysis on the early metabolic processes that influence regeneration activation.

The RNAseq analysis identified 5324 differentially expressed (DE) genes in the increased-food condition and 1830 DE genes in the penicillin treatment (S3 Table). Of these, 1416 genes are differentially expressed in both increased-food and penicillin treatment (Fig 5b and 5c; the full list in S3 Table). On these shared DE genes, we performed gene ontology (GO) enrichment analysis. The top 25 GO terms enriched in the regenerating conditions are shown in Table 1. As expected, the DE genes are enriched for cell adhesion, extracellular matrix, and migration (Table 1)—processes that accompany early stages of morphogenesis [46–48]. In addition to morphogenetic processes, the enrichment analysis recovered processes in signaling (ERK and Wnt), redox and antioxidant metabolism, and immune processes.

**Table 1:** Biological processes enriched in the regenerating conditions. The top 25 GO terms are shown here; the ordering is based on q-value. The upregulated genes only recover 24 GO terms, all shown here. See S3 Table for the remaining downregulated GO terms. A q-value cutoff of 0.05 was used for the GO enrichment analysis.

	Upregulated GO terms	Downregulated GO terms
**1**	**Glutamine family amino acid metabolic process**	Cell-substrate junction assembly
**2**	Regulation of epithelial cell migration	Cell-substrate junction organization
**3**	Cell redox homeostasis	Trachea development
**4**	Aminoglycan metabolic process	Axonemal central apparatus assembly
**5**	Epithelial cell migration	Cell-matrix assembly
**6**	Epithelium migration	Homophilic cell adhesion via plasma membrane adhesion
**7**	Tissue migration	Cell junction assembly
**8**	Cellular amino acid metabolic process	Respiratory system development
**9**	Antigen processing and presentation of peptide antigen via MHC class I	Cell-substrate adhesion
**10**	Hemolysis in other organism	Regulation of ion transport
**11**	**Glutamate metabolic process**	Regulation of focal adhesion assembly
**12**	Cytolysis in other organism	Regulation of cell-substrate junction assembly
**13**	Protein folding	Regulation of cell-substrate junction organization
**14**	**Glutamine metabolic process**	Focal adhesion assembly
**15**	**Arginine biosynthetic process**	Regulation of transmembrane transport
**16**	Regulation of ERK1/2 cascade	Regulation of ion transmembrane transport
**17**	ERK1/2 cascade	Regulation of striated muscle contraction
**18**	Sulfur compound metabolic process	Cell-cell adhesion via plasma-membrane adhesion molecules
**19**	Positive regulation of epithelial-to-mesenchymal transition	Positive regulation of Wnt signaling pathway
**20**	**Glutamine family amino acid biosynthetic process**	Axoneme assembly
**21**	**Urea cycle**	Protein localization to endoplasmic reticulum
**22**	**Urea metabolic process**	Muscle structure development
**23**	Glutathione biosynthetic process	Ventricular system development
**24**	Glycosaminoglycan metabolic process	Microtubule bundle formation
**25**		Regulation of cell-matrix adhesion

Finally the enrichment analysis also recovered, notably, many upregulated DE genes associated with arginine metabolism (Fig 5d). The enriched GO terms include the arginine biosynthetic pathway itself, also known as the urea cycle (see bolded terms in Table 1). Looking closely into the genes that drive the enrichment in arginine metabolism, we find that two genes in the pathway that catalyze the ATP-dependent steps are upregulated (*carbamoyl phosphate synthase* and *argininosuccinate synthase*; red in Fig 5e). Arginine metabolism is intertwined with that of glutamine and glutamate—the three are often grouped together because of their roles in nitrogen balance, with their metabolism adjacent to the entry of nitrogen from inorganic forms. As shown in Fig 5e, upregulated in the regenerating conditions are not only genes in the arginine cycle itself, but also multiple genes in the adjacent metabolic pathways of glutamine and glutamate.

### Arginine supplementation promotes activation of regeneration

In summary, transcriptomic analysis identifies a number of biological processes that are differentially regulated in the regenerating conditions, which can pave the way for next mechanistic studies. Of the processes enriched in the regenerating condition, we focus in this final section to further test arginine. The enrichment in arginine metabolism is interesting to us for a number of reasons. First, arginine is a non-essential amino acid that becomes essential during a period of stress, injury, and growth [49–51]—thus may plausibly be limiting in regeneration activation. Second, one of the two dominant symbionts in *Aurelia*, *Mariplasma*, which is not depleted in the regenerating conditions identified so far (Fig 2), coincidentally, is predicted to metabolize arginine (Fig 3).

**Fig 6:**
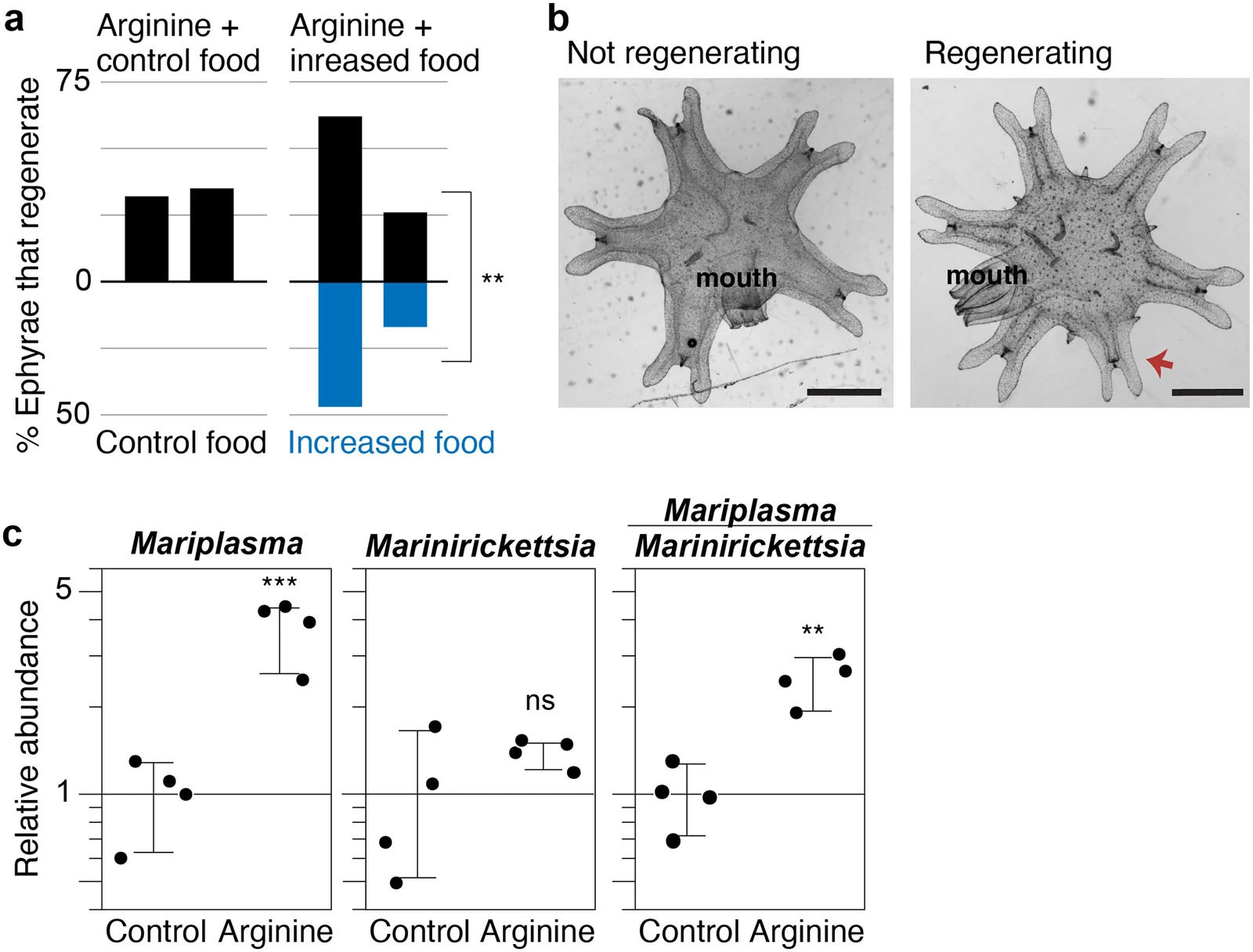
Arginine supplementation promotes regeneration. **(a-b)** Amputated ephyrae were fed control food, increased food (blue, at 3–4 times higher than control), or control food supplemented with 200 μM L-arginine (black). Regeneration was assessed two weeks after amputation. Each top and bottom pair of bars is an independent experiment, in which the two groups of animals were set up side by side (30–80 animals in each group). Statistical significance: meta-analysis. ** p < 0.001. The raw data can be found in S2 Table. **(b)** Representative ephyrae in arginine treatment. Red arrows: regenerating arms. Scale bar: 1 mm. **(c)** Relative abundance of *Marinirickettsia* and *Mariplasma* in control and arginine-supplemented condition, assessed using quantitative PCR, as described in Fig 2. Each data point (black circle) is a biological replicate (25 animals pooled). The data are plotted normalized to the average of the controls. Statistical analysis: Kruskal-Wallis test. ns: not significant. ** p < 0.001. *** p < 0.0001.

To investigate the role of arginine, we tested administering arginine to the amputated animals. Amputated animals supplemented with arginine are indeed more likely to activate appendage regeneration (Fig 6a and 6b; p < 0.001, meta-analysis, Arg vs Control). As shown in Fig 6a (left panel), 34 ± 1% of the animals activated appendage regeneration even when the control groups showed none. Arginine treatment, similar to penicillin treatment, also shows an additive effect with increasing food (Fig 6a, right panel). Finally, we assessed how arginine supplementation affects the microbiome. As expected from arginine being the fuel for *Mariplasma*, arginine supplementation markedly increases the relative abundance of *Mariplasma* (p < 0.0001, Kruskal-Wallis test, Arg vs Control), while showing much less effect on *Marinirickettsia* (Fig 6c).

## Discussion

It was previously found that increasing nutrient availability promotes activation of appendage regeneration in *Aurelia*. In this study, we find that increasing nutrient availability alters the microbiome composition. The microbiome composition in regenerating conditions can correspond to either a decrease in *Marinirickettsia* (Figs 2 and 4) or an increase in *Mariplasma* (Fig 6); both are interestingly similar in that they increase the *Mariplasma*-to-*Marinirickettsia* ratio. Since the antibiotic treatment that significantly targets *Marinirickettsia* can recapitulate regeneration induction (Fig 2), we propose that the change in microbiome composition is not a simple correlation, but meaningfully influences host regeneration. Finally, we analyzed the change in the host transcriptome with the microbiome composition that promotes regeneration, and identified, among the enriched processes, arginine metabolism. Exogenous arginine supplementation indeed promotes regeneration, even while increasing *Mariplasma* load.

How might arginine promote regeneration in *Aurelia*? In many animals, the body can normally synthesize enough arginine, except under a period of growth, stress, and injury [49–51]. In these energetically demanding periods, arginine becomes limiting, and exogenous supply of arginine from nutrients becomes essential. Arginine, and its related amino acid ornithine, plays multiple roles in tissue growth [e.g., 52,53]. Arginine is a building block of proteins and a signal that directly activates the TOR pathway, the major regulator of cell growth [54–56]. Finally, arginine can interconvert to ornithine, which is the precursor for polyamines, essential during proliferative periods and wound healing [57,58]. It will be interesting to test in the next studies whether one or more of these processes are involved in how arginine promotes regeneration.

Our data suggest that a reduction in *Marinirickettsia* is beneficial for host regeneration (Figs 2 and 4), *Marinirickettsia* is closely related to *Rickettsia*, *Neorickettsia*, and *Wolbachia* [22]; genera that house parasitic species [59–61]. Species of Rickettsiales can lack key enzymes necessary for glycolysis, and are thought to acquire pyruvate from the host [e.g., 62,63]. Consistent with many related species being parasitic, the GEM analysis in this study suggests that *Marinirickettsia* potentially competes with the host for resources, and predicts pyruvate as a top nutrient taken up by *Marinirickettsia* (Fig 3). Pyruvate is a critical ATP-producing molecule in animal cells, and glycolytic activity leading to pyruvate production can increase rapidly after injury [64,65]. Pyruvate can also play a role in regulating chromatin accessibility and transcriptional response [66]. Thus, we hypothesize that a reduction in *Marinirickettsia* may promote host regeneration by increasing the pool of available nutrients.

While our data implicate reduction in *Marinirickettsia* as beneficial for host regeneration, the role of *Mariplasma* is less clear. *Mariplasma* utilizes the arginine deiminase pathway (ADI) pathway to metabolize arginine (Fig 3), but an increase in the *Mariplasma*-to-*Marinirickettsia* ratio clearly correlates with increased regeneration (Figs 2 and 6). We are unable to test the metabolites *Mariplasma* secretes (i.e., ammonium and ornithine), since the slightest increases in their availability in water make the animals unhealthy. Since arginine supports regeneration activation (Fig 6), and *Mariplasma* metabolizes arginine to make ATP (Fig 3), one immediate hypothesis is that *Mariplasma* is competing with the host for this key metabolite. However, there is a hint from the genome analysis of a potentially intriguing role for *Mariplasma*. Examining the *Aurelia* arginine/ornithine cycle more closely, we find that the *Aurelia* genome encodes all the enzymes in the arginine cycle, except for one: *arginase* (see the blue cross in Fig 5e). The loss of *arginase* is surprising to us because the arginine/ornithine cycle is deeply conserved, whose evolutionary origin likely predates the emergence of Metazoa [67,68]. To verify the loss of *arginase* in *Aurelia*, we analyzed available cnidarian genomes, and find *arginase* in other cnidarian classes, but specifically missing in all surveyed genomes of scyphozoan jellies, the class to which *Aurelia* belongs (S3 Fig). Tantalizingly, the *Mariplasma* GEM metabolizes arginine and secretes ornithine as a by-product (the dashed box in Fig 4b). Thus, genomic analysis suggests that the *Mariplasma* ADI pathway can precisely complement a missing biochemical link in *Aurelia*, and may paradoxically support host arginine production. Indeed, as we previously surveyed (ref. 22 and references therein), while association with *Marinirickettsia* appears to be one-off, the association with *Mariplasma,* with >99% similarity in 16S rRNA sequence, recurs in *Aurelia* from multiple geographical locations, indicating the existence of an *Aurelia Mariplasma*. It will be interesting next to test if *Aurelia* and *Mariplasma* have indeed formed biochemical complementarity.

The ADI pathway is a deeply conserved pathway utilized by multiple bacterial taxa [44,69]. Thus, arginine metabolism along with the bacterial ADI pathway may be factors that can promote regeneration, potentially across species. To summarize, as the role of nutrients in promoting regeneration is increasingly identified in multiple species [1–4], the findings in this study can motivate further investigations into the role of microbiome composition in linking the nutrient environment and regeneration.

## Supporting information

S1 Fig The dominant fluxes of the *Marinirickettsia* and *Mariplasma* GEMs are robust to variation in the nutrient parameters and metabolic fluxes. Shown in all the plots are the top 25 reactions, the full reaction list can be found in S1 Table. Flux is plotted as mmol/gram dry weight/hour (mmol/gDW/h). **(a, c)** In these simulations, the GEM was solved (using FBA analysis) at varying nutrient flux. 1x nutrient flux corresponds to the solution shown in the main figures. Shown are the top 25 reactions, the full reaction list can be found in S1 Table. The dominant fluxes in the GEMs remain dominant when the nutrient fluxes into the system are varied by ten fold. **(b, d)** In these simulations, the GEMs were solved using Flux Variability Analysis (FVA), which enables identification of, for each reaction, the range of feasible fluxes that support the optimal growth. Orange circle denotes the FBA solution; blue line is the FVA range. A large flux range means that we can change the flux of that reaction, and there are other ways the network can compensate for that change while still producing the optimal growth. For instance, in the *Marinirickettsia* GEM, a major means of proton import is predicted to occur through diffusion (Htex reaction). The FVA analysis tests the effects of varying this reaction. When the Htex reaction was set to zero, protons were instead imported via the Butyrate-Proton symporter (BUTt2r reaction) and the Glycine-Proton symporter (GLYt2r reaction) (not shown). When the Htex reaction was set at maximum flux (1000 mmol/gDW/hour), this was compensated by upregulation of the D-galacturonate-Proton symporter (GALURt2r reaction), which secretes excess protons. A small flux range means that the reaction is critical for supporting an optimal growth. For instance, most fluxes in the TCA reactions in *Marinirickettsia* GEM and all ADI reactions in the *Mariplasma* GEM are highly constrained, indicating that they cannot be varied without reducing growth.
(TIFF)

S2 Fig Measurement of arm regenerate length. **(a)** Quantitation of arm length was performed in FIJI. Arm length was measured from the body margin to the rhopalium (see the red arrow). A threshold of 15% was used for calling a regenerating arm. **(b)** The length of arm regenerate is plotted as % relative to length of the intact arm. To measure the length of the intact arm, we measured the length of all five intact arms in the same individual, and then used the average value. Shown here are quantitations from 6 independent experiments, see S2 Table for more replications and statistical analysis. As clearly illustrated in Exp 3–6, penicillin-treated animals can regenerate longer arms than those stimulated by increased food availability.
(TIFF)

S3 Fig *Arginase* is missing in the scyphozoan lineage. To assess the arginine biosynthesis pathway across the cnidarian phylogeny, we analyzed available genomes of cnidarian species The arginine biosynthesis pathway is driven by five genes, all analyzed here. Black indicates the gene is present; white indicates the gene is absent. *Arginase* is present in three cnidarian classes (Anthozoa, Cubozoa, and Hydrozoa) and appears to be specifically missing in Scyphozoa. Interestingly, a different gene, *ornithine transcarbamylase* is missing from both hydrozoan genomes analyzed. The arginine biosynthesis pathway may have undergone different evolutionary sculpting in different cnidarian lineages, which can be further tested as more cnidarian genomes become available.
(TIFF)

S1 Table GEM analysis of Ca. Marinirickettsia aquamalans and Ca. Mariplasma lunae. (XLSX)

S2 Table Data from regeneration experiments. (XLSX)

S3 Table RNA sequencing data. (XLSX)
