# Supplementary material for: Microbiome modulation in regeneration in jellyfish
Source: PLoS One. 2026 Aug 21;21(8):e0355863. doi: 10.1371/journal.pone.0355863 (PMC13496418; doi:10.1371/journal.pone.0355863)
Supplement: Supplementary file 1 [file pone.0355863.s007.pdf]

Manuscript Number PONE-D-26-02629

Reviewer Comments

Lines 24–25

“In this study, we find a change in nutrient availability changes the microbiome composition.”

Consider…“In this study, we found that changes in nutrient availability alter microbiome composition.”

Lines 26–27

“Genome-scale metabolic modeling of the microbial symbionts suggests that the microbiome modulation reduces a symbiont that competes with host resources.”

Consider “…reduces the abundance of a symbiont that competes with the host for metabolic resources.” As it brings some specificity.

Lines 27–28

“Antibiotic treatment that recapitulates the microbiome shift, remarkably, strongly promotes regeneration.”

The phrase “remarkably, strongly promotes” is weak for scientific writing.

Consider “…significantly promotes regeneration.”

Lines 29–30

“Transcriptomic analysis of the host shows an upregulation of arginine metabolism.”

Clarify which pathway or genes, if possible.

For example, consider “…an upregulation of genes involved in arginine metabolism.”

Lines 30–31

“Directly administering arginine can promote appendage regeneration.”

Consider using more formal language.

“Exogenous arginine supplementation promotes ...” unless otherwise.

Lines 31–32

“These analyses suggest microbiome composition as linked with nutrient availability in influencing activation of host regeneration”

This sentence is grammatically awkward.

Note that it is the findings from the analyses that suggest it.

So, consider …“These findings suggest that microbiome composition, influenced by nutrient availability, contributes to the activation of host regeneration.”

Lines 35–36

“One limiting factor in regeneration, it is increasingly appreciated, is nutrient availability.”

The clause interrupts the sentence.

Consider …“Nutrient availability is increasingly recognized as a limiting factor in regeneration.”

Lines 37–38

“Ctenophores stop regenerating in poor nutrient environments”

Clarify experimental conditions if possible.

Consider …“Ctenophores cease regenerative processes under nutrient-limited conditions.”

Lines 63–65

The description of Aurelia anatomy and arms is clear and helpful.

However, consider adding a brief functional context (feeding, locomotion).

Lines 68–69

“While symmetrization is the robust response to injury”

Consider replacing “the robust response” with “the primary response” or “a dominant response.”

Lines 69–70

“promotes some animals to begin regenerating partial appendages”

Suggestion:

Consider clarifying the percentage or proportion if data are available and include a citation.

Lines 73–74

“Motivated by the role of nutrients, we investigated the microbiome.”

Make the hypothesis clearer by considering …“Given the role of nutrients in regeneration, we investigated whether microbiome composition mediates this relationship.”

Lines 75–76

“Microbiome not only influences nutrient metabolism, nutrients shape the microbiome”

Consider … “The microbiome not only influences nutrient metabolism, *but* nutrients also shape the microbiome.”

Lines 80–81

“Microbiome has also been linked to regeneration”

Consider …“The microbiome has also been linked to regenerative processes.”

Lines 84–86

“…assessed its potential roles in linking nutrition and regeneration activation.”

Consider “…to assess its potential role in linking nutrient availability to regeneration activation.” This is because nutrition is too broad to use.

Lines 90–92

“the microbiome of Aurelia is dominated by two taxa”

Consider adding taxonomic clarification for readability.

Example:

“the microbiome of *Aurelia* is dominated by two bacterial taxa”

Line 93

“While we do not rule out possible contributions from minor taxa”

Consider slightly tightening this sentence ... “Although minor taxa may also contribute to host physiology, our analysis focused on the two dominant taxa.”

Lines 95–96

“To probe the two taxa, we utilized the metagenome-assembled genomes…”

Consider …“To quantify these taxa, we used metagenome-assembled genomes from our previous study to design species-specific primers for quantitative PCR analysis.” This clarifies the experimental workflow

Lines 118–124

The interpretation of the microbiome shift is clear.

However, the sentence …“This downward shift… is sufficient to produce a statistically significant shift…” can better be written as …“This downward shift in *Marinirickettsia* abundance leads to a statistically significant increase in the *Mariplasma-to-Marinirickettsia* ratio.”

Lines 126–128

“The impacts of nutrient availability on the microbiome composition motivated us to build genome-scale metabolic models”

This transition is good, but consider ending the sentence with…“of the two dominant bacterial taxa.”

Lines 129–130

“A GEM translates the genome into a network of metabolic reactions”

Consider “…translate *genomic information* into networks of metabolic reactions.”

Lines 131–133

To remove redundancy in …“The small genomes and consequently reduced numbers of genes mean that there are few alternative metabolic networks”

Consider …“The small genome sizes (0.4 Mb for *Mariplasma* and 1 Mb for *Marinirickettsia*) limit the number of alternative metabolic pathways encoded.”

Lines 139–141

The host competition hypothesis is interesting but should be phrased cautiously.

Consider rephrasing from “this predicts a significant competition with the host” to “suggesting potential metabolic competition with the host.”

Lines 183–186

“Motivated by the qPCR measurements and the GEM analysis…”

Consider beginning with …“Based on the qPCR results and GEM predictions, ….”

Lines 191–193

Consider paraphrasing “…performed amputation experiments” to “…performed appendage amputation experiments”

Lines 198–199

Consider paraphrasing “…penicillin-treated animals can regenerate longer arms” to “…Penicillin-treated animals also regenerated significantly longer appendages.”

Lines 242–244

Avoid the subjective wording “strikingly”

Consider “Notably”

Lines 277–284

Consider reporting exact percentages if available, which is much better than “more than a quarter of the animals.”

Overall Recommendation

The study demonstrates a clear experimental progression, with strong integration of microbiome and host biology. It employs multiple independent experimental approaches and presents a logical narrative that ultimately leads to the identification of the arginine mechanism.

Improvement needed:

Several revisions are recommended to improve the clarity and scientific presentation of the manuscript.

First, grammatical errors and incomplete sentences should be corrected throughout the text to enhance readability.

In addition, some figure legend material appears within the Results section and should be relocated to the appropriate figure captions to maintain proper manuscript structure.

The Results section should primarily present the findings, with interpretive statements minimized and reserved for the Discussion, where they can be more appropriately contextualized.

Finally, statistical analyses should be consistently and clearly reported, including the tests used and significance thresholds, to ensure transparency and reproducibility.

Addressing these issues will strengthen the clarity, organization, and overall quality of the manuscript.
