# Supplementary material for: Microbiome modulation in regeneration in jellyfish
Source: PLoS One. 2026 Aug 21;21(8):e0355863. doi: 10.1371/journal.pone.0355863 (PMC13496418; doi:10.1371/journal.pone.0355863)
Supplement: Supplementary file 2 [file pone.0355863.s009.pdf]

Response to Reviewers

Overall Recommendation

The study demonstrates a clear experimental progression, with strong integration of microbiome and host biology. It employs multiple independent experimental approaches and presents a logical narrative that ultimately leads to the identification of the arginine mechanism.

Improvement needed:
Several revisions are recommended to improve the clarity and scientific presentation of the Manuscript. First, grammatical errors and incomplete sentences should be corrected throughout the text to enhance readability.
Response: We thank the reviewers for the detailed line-by-line suggestions, and have addressed each point. In addition, we have systematically gone through the manuscript again to remove grammatical errors.

In addition, some figure legend material appears within the Results section and should be relocated to the appropriate figure captions to maintain proper manuscript structure.
Response: We have now revised the manuscript to follow the figure and figure legend placement according to the PLOS ONE formatting guidelines.

The Results section should primarily present the findings, with interpretive statements minimized and reserved for the Discussion, where they can be more appropriately contextualized.
Response: We have gone through the Results section and moved the interpretive statements to the Discussion.

Finally, statistical analyses should be consistently and clearly reported, including the tests used and significance thresholds, to ensure transparency and reproducibility. Addressing these issues will strengthen the clarity, organization, and overall quality of the manuscript.
Response: We have gone through the manuscript and made sure, where statistical tests are employed, the p-values, test, and comparison performed are described.

We sincerely thank the Reviewers and the Editor for the feedback. We believe that the suggested changes have greatly improved the clarity and readability of the manuscript. All changes are detailed in these next sections, and tracked in the manuscript.

Reviewers' comments

1. Is the manuscript technically sound, and do the data support the conclusions?
Reviewer #1: Yes
Reviewer #2: Yes

2. Has the statistical analysis been performed appropriately and rigorously?
Reviewer #1: I Don't Know
Reviewer #2: Yes

3. Have the authors made all data underlying the findings in their manuscript fully available?
Reviewer #1: Yes
Reviewer #2: Yes

4. Is the manuscript presented in an intelligible fashion and written in standard English?
Reviewer #1: Yes
Reviewer #2: Yes

5. Review Comments to the Author

Reviewer #1

This study addresses an interesting and timely question: how nutritional conditions and microbiome modulation relate to regeneration in Aurelia coerulea. The experimental approach has clear potential, and several sections are well written. However, the manuscript currently has important weaknesses in framing and focus, especially in the Introduction **(R1P1)**; methodological reproducibility some key details are missing **(R1P2)**; and structure, and the Discussion is too short and under-referenced **(R1P3)**. Strengthening these areas would greatly improve clarity, rigor, and impact of this work.

We thank Reviewer #1 for the feedback and have carefully made major edits to the Introduction, description of methodology, and Discussion to improve clarity. These changes are outlined below.

R1P1. The manuscript currently has important weaknesses in framing and focus, especially in the Introduction.
We have refocused the framing in the Introduction. We previously went back and forth between motivating the importance of nutrients vs. the importance of the microbiome in regeneration. In this revised version, we focused on investigating the role of the microbiome in regeneration in the context of varying nutrients. We revised the transition sentences more, and reordered the last two paragraphs. We hope this reads more focused now, and welcome any further suggestions. For convenience, the revised Introduction (line 37–77) is included below, with the new text in blue, and the original text in black.

Introduction

Recovering injured organs is energetically demanding. It is increasingly appreciated that a ~~One~~ limiting factor in regeneration ~~increasingly appreciated,~~ is nutrient availability. Ctenophores ceases~~stop~~ regenerativ~~eng~~ processes under ~~in~~ nutrient-limited ~~poor~~ nutrient conditions [1]. Salamanders reared in low nutrient conditions show~~ed~~ substantially reduced limb regeneration [2].

Nutrients are not only necessary for supporting regeneration; increasing nutrient availability can, remarkably, promote activation of regeneration in poorly regenerating systems. In jellyfish, increasing nutrient availability promotes activation of appendage regeneration [3]. In tadpoles, which stop regenerating their tails during the refractory period, increaseding feeding restores tail regeneration [4]. In adult mice, which do not normally regrow digits from proximal amputations, increasing nutrients promotes activation of digit regrowth [3]. Although the regeneration activated is not perfect and not all animals activate regeneration, these studies identify nutrients as a limiting factor in poorly regenerating systems. Given Motivated by the role of nutrients in regeneration, we investigated whether the microbiome plays a role in mediating this relationship.

The microbiome can profoundly influence the host energy nutrient metabolism [5]. The microbiome can aid the breakdown of nutrients, produce molecules otherwise not available to the host, and increase amino acid availability [e.g. 6–10]. From flies to mice, the microbiome can compensate for undernutrition and rescue growth [11,12]. Microbiome not only influences nutrient metabolism, but nutrients also shape the microbiome (12,13). Change in diet can alter the microbiome composition as rapidly as within 24 hours in humans (14). Moreover, some of the effects of nutrients are attributed to their impact on the microbiome. For instance, a diet high in fermented food reduces inflammatory markers, in part through modifying the microbiome composition (15). In parallel, the microbiome has also been linked to regenerativeeon processes [13]. In planarians, a pathogenic shift in the microbiome inhibits regeneration [14]. In mice, taxa-specific signals from the gut influence regeneration processes in remote muscle and liver [15]. In frog tadpoles, antibiotic treatment inhibits tail regeneration, which can be rescued by supplying lipopolysaccharides [16]. While these studies connected microbiome and regeneration, few have looked into this connection in the context of varying nutrients. In this study, we began exploring the connections between nutrients, microbiome, and regeneration.

The role of the nutrients on influencing regeneration activation is conserved in *Aurelia coerulea*. To investigate this, we used the moon jellyfish *Aurelia coerulea* as a model system. *Aurelia* is well-suited for this study because its regeneration responses vary across its life cycle (Fig 1a), allowing us to assay regeneration induction. In the sessile stage, similar to other cnidarian polyps such as hydras and sea anemones, *Aurelia* polyps readily regenerate body parts [17,18]. However, in the free-swimming stages, *Aurelia* ephyrae and medusae show limited regeneration [19,20]. This is clearly demonstrated in the ephyra (Fig 1b), whose eight discrete swimming appendages, called arms, facilitate tracking of regeneration, or the lack of it. These arms function in swimming and feeding; they contract synchronously to produce fluid flow that propels the animal and captures prey [21]. In response to amputation (Fig 1c), *Aurelia* ephyrae rapidly reorganize the remaining body parts and arms, and regain radial symmetry (Fig 1d). The recovery of radial symmetry facilitates propulsion and feeding, and ultimately, continued survival [19]. While symmetrization is the primaryrobust response to injury, as noted above, it was a previous study found that increasing nutrient availability promotes some ~40% of animals to begin regenerating partial appendages (ref. 3; reproduced here in Figure 1e and again later in Figure 4b). This observation provides a window to study in a basal animal lineage the conserved influence of nutrients in regeneration. In addition to the established connection between nutrient and regeneration, the microbiome of *Aurelia* has been characterized. A recent study found that the *Aurelia* microbiome is dominated by two bacterial taxa [22]. Therefore, *Aurelia* offers a low complexity microbiome system to interrogate how the microbiome and nutrients interact to affect regeneration in a poorly regenerating animal.

R1P2. Methodological reproducibility some key details are missing.
We appreciate the Reviewer for catching this. We have added more key details to the Methods to better ensure that the experimental and analysis methodology can be reproduced by other labs. The new additions are listed below. We welcome further suggestions from the Reviewer.

Line 105–127: **Amputation experiments and treatment.** … fitted with a #17 blade following methods described previously (3). Under a dissecting microscope, ephyrae held in a petri dish were amputated by cutting across the body to remove three arms (Fig 1c). Two-day old ephyrae were used for the amputation experiments. … For control, low-food condition, ephyrae were fed ~5 rotifers per animal every other day. For the increased food condition, ephyrae were fed ~40 rotifers per animal. … For penicillin treatment, penicillin was added to the water to give a final concentration of 100 U/mL …(G-Biosciences, RC-741). For arginine experiments, L-arginine methyl ester dihydrochloride was added to the water to give a final concentration of 200 µM (Chem-IMPEX International Inc., 03022). Penicillin and arginine treatments were performed with control or increased food condition, as specified in the main text. Water …

Line 152–177: **Quantitative PCR.** … DNA extraction was performed using the DNeasy Blood and Tissue Kit (Qiagen, 69504), with 3 hour proteinase K treatment. qPCR was performed …

To calculate the relative abundance of the bacteria, *Ct* values were first converted to raw DNA quantities and corrected to account for primer efficiency using the formula:

$$\textit{DNA quantity} = 10^{((\text{Ct value} - \textit{intercept})/\textit{-slope})}$$

The DNA quantity was then normalized by the host *Sox1* gene, to account for loading variability. Subsequently, the *Sox1*-normalized DNA quantity was normalized by the mean of the control data. These are the relative bacterial abundances plotted in the main figures. *Mariplasma*-to-*Marinirickettsia* ratios were calculated by taking the ratio of *Mariplasma* and *Marinirickettsia* relative abundance within each biological replicate. To compute statistical significance, the non-parametric Kruskal-Wallis test was used.

Line 179–197: **Reconstruction of the genome-scale metabolic models (GEMs).** … model reconstruction of the *Mariplasma* and *Marinirickettsia* genomes (NCBI Accession PRJNA975886) was performed with CarveMe (version 1.3.0) (25) … optimum growth conditions (as determined with *cobra_model.optimize()*, where *cobra_model* is the name of the model input) … knock out simulation (using *cobra_model.reactions.reactionID.knock_out(), where cobra_model* is the name of the model *reactionID* is the name of the reaction); and (3) The reaction …

Line 199–218: **RNA sequencing and analysis.** … RNA extraction was performed using the RNeasy Plus Mini kit (Qiagen, 74104), with the following modifications. ~~Briefly,~~ RLT buffer containing β-mercaptoethanol was added to the frozen samples and the samples were homogenized on ice. Homogenized samples were centrifuged ….

Line 220–226: **Presence or absence of *arginase*.** … annotated genomes. The cnidarian genomes were retrieved from the repositories specified in the source papers. For *Hydra*, protein models from version 2.0 were retrieved from the NHGRI repository (https://research.nhgri.nih.gov/hydra/). Gene models…

R1P3. The Discussion is too short and under-referenced.
We have added more points for Discussion, and strengthened the references (the new references are track changed). We have also edited the first paragraph of the Discussion to reflect the refocusing that the Reviewer suggested in point 1 above. The revised Discussion (line 426–487) is included below, we welcome any further suggestions. We thank the Reviewer, and believe the Discussion and Introduction sections have been strengthened by the recommended major edits.

Discussion

It was previously found that increasing nutrient availability promotes activation of appendage regeneration in *Aurelia*. In this study, we find that increasing nutrient availability ~~the conditions that promote host regeneration (high nutrient availability, antibiotic treatment, and arginine supplementation) also~~ alters the microbiome composition. The microbiome composition in regenerating conditions can correspond to either a decrease in *Marinirickettsia* (Fig 2, 4) or an increase in *Mariplasma* (Fig 6); both are interestingly similar in that they increase the *Mariplasma*-to-*Marinirickettsia* ratio. Since the antibiotic treatment that significantly targets *Marinirickettsia* can recapitulate regeneration induction (Fig 2), we propose that the change in microbiome composition is not a simple correlation, but meaningfully influences host regeneration. Finally, we analyzed the change in the host transcriptome with the microbiome composition that promotes regenerati~~ng~~on ~~conditions~~, and identified, among the enriched processes, arginine metabolism. ~~Directly administering~~ Exogenous arginine supplementation indeed promotes regeneration even while increasing *Mariplasma* load.

How might arginine promote regeneration in *Aurelia*? In many animals, the body can normally synthesize enough arginine, except under a period of growth, stress, and injury [49–51]. In these energetically demanding periods, arginine becomes limiting, and exogenous supply of arginine from nutrients becomes essential. Arginine, and its related amino acid ornithine, plays multiple roles in tissue growth [e.g. 52,53]. Arginine is a building block of proteins and a signal that directly activates the TOR pathway, the major regulator of cell growth [54–56]. Finally, arginine can interconvert to ornithine, which is the precursor for polyamines, essential during proliferative periods and wound healing [57–58]. It will be interesting next to test in the next studies whether one or more of these processes are involved in how arginine promotes regeneration.

Our data suggest that a reduction in *Marinirickettsia* is beneficial for host regeneration (Fig 2,4)~~,~~ *Marinirickettsia* is closely related to *Rickettsia*, *Neorickettsia*, and *Wolbachia* [22]; genera that house parasitic species [59–61]. Species of Rickettsiales can lack key enzymes necessary for glycolysis, and are thought to acquire pyruvate from the host [e.g., 62,63]. Consistent with many related species being parasitic, the GEM analysis in this study suggests that *Marinirickettsia* potentially competes with the host for resources, and predicts pyruvate as a top nutrient taken up by *Marinirickettsia* (Fig 3). Pyruvate is a critical ATP-producing molecule in animal cells, and glycolytic activity leading to pyruvate production can increase rapidly after injury [64,65]. Pyruvate can also play a role in regulating chromatin accessibility and transcriptional response [66]. Thus, we hypothesize that a reduction in *Marinirickettsia* may promote host regeneration by increasing the pool of available nutrients.

While our data implicate reduction in *Marinirickettsia* as beneficial for host regeneration, the role of *Mariplasma* is less clear. *Mariplasma* utilizes the arginine deiminase pathway (ADI) pathway to metabolize arginine (Fig 3), but an increase in the *Mariplasma*-to-*Marinirickettsia*

ratio clearly correlates with increased regeneration (Fig 2, 6). We ~~were~~are unable to ~~directly~~ test the metabolites *Mariplasma* secretes,~~ (~~ammonium and ornithine~~)~~, since slightest increases in their availability in water made the animals unhealthy. Since arginine supports regeneration activation (Fig 6), and *Mariplasma* metabolizes arginine to make ATP (Fig 3), one immediate hypothesis is that *Mariplasma* is competing with the host for this key metabolite. However, there is a hint from the genome analysis of a potentially intriguing role for *Mariplasma*. Examining the *Aurelia* arginine/ornithine cycle more closely, we find that the *Aurelia* genome encodes all the enzymes in the arginine cycle, except for one: *arginase* (see the blue cross in Fig 5e). The loss of *arginase* is surprising to us because the arginine/ornithine cycle is deeply conserved, whose evolutionary origin likely predates the emergence of Metazoa [67,68]. To verify the loss of *arginase* in *Aurelia*, we analyzed available cnidarian genomes, and find *arginase* in other cnidarian classes, but specifically missing in all surveyed genomes of scyphozoan jellies, the class to which *Aurelia* belongs (Fig S3). Tantalizingly, the *Mariplasma* GEM metabolizes arginine and secretes ornithine as a by-product (the dashed box in Fig 4b). Thus, genomic analysis suggests that the *Mariplasma* ADI ~~a microbial~~ pathway can precisely complement a missing biochemical link in *Aurelia*, and may paradoxically support host arginine production. Indeed, as we previously surveyed (ref. 22 and references therein), while association with *Marinirickettsia* appears to be one-off, the association with *Mariplasma,* with >99% similarity in 16S rRNA sequence, recurs in *Aurelia* from multiple geographical locations, indicating the existence of an *Aurelia Mariplasma*. It will be interesting next to test if *Aurelia* and *Mariplasma* have indeed formed biochemical complementarity.

The ADI pathway is a deeply conserved pathway utilized by multiple bacterial taxa [44,69]. Thus, arginine metabolism along with the bacterial ADI pathway may be factors that can promote regeneration, potentially across species. To summarize, as the role of nutrients in promoting regeneration is increasingly identified in multiple species [1–4], the findings in this study can motivate further investigations into the role of microbiome composition in linking the nutrient environment and regeneration.

<u>Reviewer #2</u>

The manuscript presents an interesting and well-designed study which examined the relationship between nutrient availability, microbiome composition, and regeneration in Aurelia.

The integration of microbiome characterization, genome-scale metabolic modeling, host transcriptomic analysis, and functional experiments (antibiotic treatment and arginine supplementation) provides a strong and multifaceted approach to investigating the biological mechanisms underlying regeneration.

The experimental progression is logical and the findings contribute valuable insight into potential interactions between microbial symbionts and host metabolic pathways in regenerative processes.

However, several revisions are recommended to improve the clarity, structure, and presentation of the manuscript. First, grammatical errors and incomplete sentences should be corrected throughout the text to improve readability and precision (**R2P1**). The Results section would also benefit from focusing primarily on the presentation of experimental findings, with interpretive statements minimized and reserved for the Discussion where they can be more fully contextualized (**R2P2**). Finally, statistical analyses should be reported consistently and clearly, including the statistical tests used, significance thresholds, and comparisons performed, to ensure transparency and reproducibility (**R2P3**).

R2P1. Grammatical errors and incomplete sentences should be corrected throughout the text to improve readability and precision.
We are grateful to Reviewer 2 for taking the time to provide the line-by-line suggestions included in the PDF file accompanying the review. This was incredibly helpful, and we have followed most of the suggested revisions (see Line-by-line section below). Additionally, we went through the manuscripts systemically to detect and remove grammatical errors and incomplete sentences. All changes in the revised manuscript are tracked.

R2P2. The Results section would also benefit from focusing primarily on the presentation of experimental findings, with interpretive statements minimized and reserved for the Discussion where they can be more fully contextualized.
We agree with Reviewer 2 that the Result section should strictly describe the results of the experimental or modeling analyses. We went through each section of the Results, and identified the following interpretive sentences in the GEM section. The sentences have either been revised to make them strictly summarize the analysis results, or moved to the Discussion section. The changes are outlined below, with the new text in blue, and the original text in black.

The following interpretive sentences have been revised to remove speculation (now line 301-304):
“Thus, the GEM analysis ~~suggests~~ shows that the bacterium that is depleted in the regenerating condition, *Marinirickettsia*, is predicted to sequester high-energy metabolites from the host. On the other hand, *Mariplasma*, which remains unaffected in the regeneration condition, ~~may not be energetically detrimental to the host.~~ is predicted to sequester fewer metabolites from the host”

The following interpretive sentences (underlined) have been moved to the Discussion:

Original lines 140–141: "… to fuel its TCA cycle, *Marinirickettsia* GEM uptakes pyruvate (Figure 3b). Since pyruvate is also a critical ATP-producing molecule in animal cells, this predicts a significant competition with the host. In addition to pyruvate …"

Original line 149–152: " … including the majority of acids and cofactors (Table S1). *Marinirickettsia* is closely related to *Rickettsia* and *Neorickettsia*, genera that house many parasitic species. Consistent with many related species being parasitic, the GEM analysis suggests that *Marinirickettsia* competes with the host for resources.

In contrast to *Marinirickettsia* GEM, … "

We now discuss these points in a new paragraph in the Discussion, line 444–457:
"...proliferative periods and wound healing [57,58]. It will be interesting to test in the next studies whether one or more of these processes are involved in how arginine promotes regeneration.

Our data suggest that a reduction in *Marinirickettsia* is beneficial for host regeneration (Fig 2, 4). *Marinirickettsia* is closely related to *Rickettsia*, *Neorickettsia*, and *Wolbachia*; genera that house parasitic species [59–61]. Species of Rickettsiales can lack key enzymes necessary for glycolysis, and are thought to acquire pyruvate from the host [e.g. 62,63]. Consistent with many related species being parasitic, the GEM analysis suggests that *Marinirickettsia* potentially competes with the host for resources, and predicts pyruvate as a top nutrient taken up by *Marinirickettsia* (Fig 3). Pyruvate is a critical ATP-producing molecule in animal cells, and glycolytic activity leading to pyruvate production can increase rapidly after injury [64,65]. Pyruvate can also play a role in regulating chromatin accessibility and transcriptional response [66]. Thus, we hypothesize that a reduction in *Marinirickettsia* may promote host regeneration by increasing the pool of available nutrients.

While our data implicate reduction in *Marinirickettsia* as beneficial for host regeneration ..."

R2P3. Statistical analyses should be reported consistently and clearly, including the statistical tests used, significance thresholds, and comparisons performed, to ensure transparency and reproducibility.
We went through the Results and Supplement sections to ensure that all statistical analyses are consistently reported. The following changes have been made.

Line 261: "… the *Mariplasma*-to-*Marinirickettsia* ratio (Fig 2c, by a mean of two-fold; $p<0.001$, Kruskal-Wallis test, Pen vs Control). These measurements show that …"

Line 317–318: Penicillin treatment strongly promotes regeneration (Fig 4b,c, and see more replications in Table S2; $p<0.0001$, meta-analysis, Pen vs Control): 65–75% of penicillin-treated…"

Line 416: "… arginine are indeed more likely to activate appendage regeneration (Fig 6a,b; $p<0.001$, meta-analysis, Arg vs Control). As shown in Figure 6a …"

Line 422: "… supplementation significantly increases the relative abundance of *Mariplasma* ($p<0.0001$, Kruskal-Wallis test, Arg vs Control), while showing much less …"

Line 351–352, Fig 5 legend: " **(b-c)** Differentially expressed (DE) genes were determined using DEseq analysis~~. with cutoff.~~ Statistically significant DE genes are those with p-value <0.05 in both pairwise comparisons (Increased food vs Control and Pen vs Control). **(c)**…"

Line 355–356, Fig 5 legend: "**(d)**… plotted using Revigo. A q-value cutoff of 0.05 was used for the GO enrichment analysis. Circle size…"

Line 376–377, Table 1 legend: "... See Table S3 for the remaining downregulated GO terms. A q-value cutoff of 0.05 was used for the GO enrichment analysis."

Line-by-line comments

We thank the Reviewer for these line-by-line comments. We have adopted most of the suggestions, and provide justification for a few instances where we did not. We are open to additional feedback if our justification is not acceptable.

Suggestions for the Abstract section

LBLP1. Lines 24–25: "In this study, we find a change in nutrient availability changes the microbiome composition." Consider…"In this study, we found that changes in nutrient availability alter microbiome composition."

LBLP2. Lines 26–27: "Genome-scale metabolic modeling of the microbial symbionts suggests that the microbiome modulation reduces a symbiont that competes with host resources." Consider "…reduces the abundance of a symbiont that competes with the host for metabolic resources." As it brings some specificity.

LBLP3. Lines 27–28: "Antibiotic treatment that recapitulates the microbiome shift, remarkably, strongly promotes regeneration." The phrase "remarkably, strongly promotes" is weak for scientific writing. Consider "…significantly promotes regeneration."

LBLP4. Lines 29–30: "Transcriptomic analysis of the host shows an upregulation of arginine metabolism." Clarify which pathway or genes, if possible. For example, consider "…an upregulation of genes involved in arginine metabolism."

LBLP5. Lines 30–31: Consider using more formal language. "Exogenous arginine supplementation promotes ..." unless otherwise.

LBLP6. Lines 31–32: "These analyses suggest microbiome composition as linked with nutrient availability in influencing activation of host regeneration" This sentence is grammatically awkward. Note that it is the findings from the analyses that suggest it. So, consider …"These findings suggest that microbiome composition, influenced by nutrient availability, contributes to the activation of host regeneration."

We have incorporated all these suggestions (LBLP1-LBLP6) in the revised Abstract (now lines 23–34):

The moon jelly *Aurelia coerulea* does not typically regenerate appendages. A previous study found that increasing nutrient availability promotes the activation of appendage regeneration. In this study, we ~~find a~~ found that changes in nutrient availability ~~changes the~~ alter microbiome composition. Genome-scale metabolic modeling of the microbial symbionts suggests that the microbiome modulation reduces the abundance of a symbiont that competes with the host for

metabolic resources. Antibiotic treatment that recapitulates the microbiome shift, remarkably, strongly significantly promotes regeneration. Transcriptomic analysis of the host shows an upregulation of genes involved in the arginine metabolism biosynthetic pathway, including *argininosuccinate synthase* and *carbamoyl phosphate synthase*. Directly administering Exogenous arginine can supplementation promotes appendage regeneration. These analyses findings suggest that microbiome composition, as linked with influenced by nutrient availability, in influencing contributes to the activation of host regeneration, and identify a role for arginine in promoting regeneration.

Suggestions for the Introduction section

LBLP7. Lines 37–38: "One limiting factor in regeneration, it is increasingly appreciated, is nutrient availability." The clause interrupts the sentence. Consider …"Nutrient availability is increasingly recognized as a limiting factor in regeneration."

The sentence has been revised (now line 37): "It is increasingly appreciated that a limiting factor in regeneration is nutrient availability. … "

LBLP8. Lines 38–39: "Ctenophores stop regenerating in poor nutrient environments" Clarify experimental conditions if possible. Consider …"Ctenophores cease regenerative processes under nutrient-limited conditions."

The sentence has been revised as suggested (now line 38–39): "Ctenophores stop cease regeneratingve processes under in poor nutrient-limited environments conditions [1]."

LBLP9. Lines 65–67: The description of Aurelia anatomy and arms is clear and helpful. However, consider adding a brief functional context (feeding, locomotion).

We have added a brief description of the functional context (lines 65–67):

"...the ephyra (Fig 1b), whose eight discrete swimming appendages, called arms, facilitate tracking of regeneration, or the lack of it. These arms function in swimming and feeding; they contract synchronously to produce fluid flow that propels the animal and captures prey [21]. In response to amputation…"

LBLP10. Lines 68–69: "While symmetrization is the robust response to injury". Consider replacing "the robust response" with "the primary response" or "a dominant response."

The sentence has been revised (now line 70): "While symmetrization is the primary robust response to injury…"

LBLP11. Lines 69–70: "promotes some animals to begin regenerating partial appendages" Suggestion: Consider clarifying the percentage or proportion if data are available and include a citation.

We have revised the sentence as suggested (now lines 71): "... a previous study found that increasing nutrient availability promotes some ~40% of animals to begin regenerating partial appendages (ref. 3; reproduced here in Fig 1e and again later in Fig 4b). …"

LBLP12. Lines 73–74: "Motivated by the role of nutrients, we investigated the microbiome." Make the hypothesis clearer by considering …"Given the role of nutrients in regeneration, we investigated whether microbiome composition mediates this relationship."

We have revised the sentence as suggested (now lines 46–47):“ Given the role of nutrients in regeneration, we investigated ~~the~~ whether the microbiome plays a role in mediating this relationship.

LBLP13. Lines 75–76: “Microbiome not only influences nutrient metabolism, nutrients shape the microbiome”. Consider … “The microbiome not only influences nutrient metabolism, *but* nutrients also shape the microbiome.”
This sentence has now been removed as a result of the major edits in the Introduction suggested by Reviewer 1.

LBLP14. Lines 80–81: “Microbiome has also been linked to regeneration.” Consider … “The microbiome has also been linked to regenerative processes.”
We have revised the sentence accordingly (now lines 51–52): “the m~~M~~icrobiome has also been linked to regenerati~~on~~ve processes [13].

LBLP15. Lines 84–86: “…assessed its potential roles in linking nutrition and regeneration activation.” Consider “… to assess its potential role in linking nutrient availability to regeneration activation.” This is because nutrition is too broad to use.
This sentence has now been removed as a result of the major edits in the Introduction suggested by Reviewer 1.

<u>Suggestions for the Results section</u>

LBLP16. Lines 90–92: “the microbiome of Aurelia is dominated by two taxa”. Consider adding taxonomic clarification for readability. Example: “the microbiome of *Aurelia* is dominated by two bacterial taxa”
We have revised the sentence accordingly (now lines 229): “As characterized in previous work (22), the microbiome of *Aurelia* is dominated by two bacterial taxa, …”

LBLP17. Line 93: “While we do not rule out possible contributions from minor taxa.” Consider slightly tightening this sentence ... “Although minor taxa may also contribute to host physiology, our analysis focused on the two dominant taxa.”
We have revised the sentence accordingly (now lines 232–233): “~~While we do not rule out possible contributions from~~ Although minor taxa may also contribute to host physiology, ~~we~~ our analysis focused ~~here~~ on the dominant taxa.”

LBLP18. Lines 95–96: “To probe the two taxa, we utilized the metagenome-assembled genomes…” Consider …“To quantify these taxa, we used metagenome-assembled genomes from our previous study to design species-specific primers for quantitative PCR analysis.” This clarifies the experimental workflow.
We have revised the sentence accordingly (now lines 234–235): “To ~~probe the two~~ quantify these taxa, we utilized the metagenome-assembled genomes ~~constructed in~~ from our previous study [22] to design species-specific primers~~, and performed~~ for quantitative PCR ~~to measure the relative abundance of the bacteria~~ analysis (Fig 2).”

LBLP19. Lines 118–124: The interpretation of the microbiome shift is clear. However, the sentence …“This downward shift… is sufficient to produce a statistically significant shift…” can

better be written as …"This downward shift in *Marinirickettsia* abundance leads to a statistically significant increase in the *Mariplasma-to-Marinirickettsia* ratio."
We have revised the sentence accordingly (now lines 259–260): "This downward shift in *Marinirickettsia* abundance ~~is sufficient to produce~~ leads to a statistically significant ~~shift in the microbiome composition,~~ increase~~ing~~ in the *Mariplasma*-to-*Marinirickettsia* ratio (Fig 2c, by a mean of two-fold)."

LBLP20. Lines 126–128: "The impacts of nutrient availability on the microbiome composition motivated us to build genome-scale metabolic models" This transition is good, but consider ending the sentence with…"of the two dominant bacterial taxa."
We have revised the sentence accordingly (now lines 266): "The impacts of nutrient availability on the microbiome composition motivated us to build genome-scale metabolic models (GEMs) of the two dominant bacterial taxa (Fig 3)."

LBLP21. Lines 129–130: "A GEM translates the genome into a network of metabolic reactions." Consider "…translate *genomic information* into networks of metabolic reactions."
We have revised the sentence accordingly (now lines 268): "A GEM translates the genom~~e~~ic information into a network of metabolic reactions,..."

LBLP22. Lines 131–133: To remove redundancy in …"The small genomes and consequently reduced numbers of genes mean that there are few alternative metabolic networks." Consider …"The small genome sizes (0.4 Mb for *Mariplasma* and 1 Mb for *Marinirickettsia*) limit the number of alternative metabolic pathways encoded."
We agree with the reviewer that in the context of bacteria, a small genome means fewer genes. However, since this is not necessarily true for non-bacterial contexts, we included this redundancy to ensure that the broader audience understands this link, and would prefer to leave this sentence as is.

LBLP23. Lines 139–141: The host competition hypothesis is interesting but should be phrased cautiously. Consider rephrasing from "this predicts a significant competition with the host" to "suggesting potential metabolic competition with the host."
We have now revised this sentence and, as suggested in Point 2 by Reviewer 2, moved this interpretive sentence to the Discussion section (lines 451–452):
".... Consistent with many related species being parasitic, the GEM analysis in this study ~~indeed~~ suggests that *Marinirickettsia* potentially competes with the host for resources, …"

LBLP24. Lines 183–186: "Motivated by the qPCR measurements and the GEM analysis…" Consider beginning with …"Based on the qPCR results and GEM predictions, …."
We have revised the text accordingly (now line 308): "~~Motivated by~~ Based on the qPCR ~~measurements~~ results and the GEM ~~analysis~~ predictions, we tested …"

LBLP25. Lines 191–193: Consider paraphrasing "…performed amputation experiments" to "…performed appendage amputation experiments"
Since we are amputating across the body (see the amputation scheme in Fig 1c, included below for convenience), we have carefully avoided the phrase "appendage amputation".

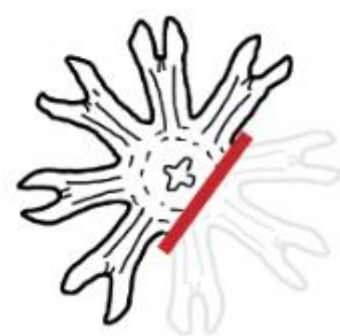

LBLP26. Lines 198–199: Consider paraphrasing "…penicillin-treated animals can regenerate longer arms" to "…Penicillin-treated animals also regenerated significantly longer appendages."
We want to communicate to the readers that while some penicillin-treated animals regenerated longer arms, some do not. We therefore think the sentence as is captures this nuance.

LBLP27. Lines 242–244: Avoid the subjective wording "strikingly". Consider "Notably"
We have revised the sentence accordingly (now line 379): "Finally the enrichment analysis also recovered, ~~strikingly~~ notably, many upregulated DE genes associated with arginine metabolism…"

LBLP28. Lines 277–284: Consider reporting exact percentages if available, which is much better than "more than a quarter of the animals."
We have revised the sentence accordingly (now line 417): "As shown in Fig 6a (left panel), 34±1%~~more than a quarter~~ of the animals activated appendage…"
